# Epigenetics of oropharyngeal squamous cell carcinoma: opportunities for novel chemotherapeutic targets

**DOI:** 10.1186/s40463-017-0185-3

**Published:** 2017-01-31

**Authors:** Cameron Lindsay, Hadi Seikaly, Vincent L. Biron

**Affiliations:** Faculty of Medicine and Dentistry, Department of Surgery, Division of Otolaryngology-Head and Neck Surgery, University of Alberta, 1E4.34 WMC, 8440 112 Street, Edmonton, AB T6G 2B7 Canada

**Keywords:** Oropharyngeal cancer, Human papillomavirus, Squamous cell carcinoma, Epigenetics, Chemotherapy

## Abstract

Epigenetic modifications are heritable changes in gene expression that do not directly alter DNA sequence. These modifications include DNA methylation, histone post-translational modifications, small and non-coding RNAs. Alterations in epigenetic profiles cause deregulation of fundamental gene expression pathways associated with carcinogenesis. The role of epigenetics in oropharyngeal squamous cell carcinoma (OPSCC) has recently been recognized, with implications for novel biomarkers, molecular diagnostics and chemotherapeutics. In this review, important epigenetic pathways in human papillomavirus (HPV) positive and negative OPSCC are summarized, as well as the potential clinical utility of this knowledge.

This material has never been published and is not currently under evaluation in any other peer-reviewed publication.

## Background

The field of epigenetics is defined as the study of heritable changes in gene expression without alteration of the DNA sequence. Epigenetic regulation has been implicated in multiple phenomena in both plants and animals. These include embryonic development, cell differentiation, imprinting, X chromosome inactivation, and various other gene expression patterns [1–3]. Aberrations within the epigenome have been implicated in several human diseases and most types of cancer [2, 4–8].

Epigenetic changes influence tumor development, proliferation, metastasis, and resistance to chemoradiotherapies [2, 4–7]. The primary mechanisms of epigenetic carcinogenesis involve DNA methylation, histone modifications, small and non-coding RNAs (ncRNA), which ultimately orchestrate complex gene regulatory pathways [1, 9–12]. Over the past decade, various aspects of these epigenetic processes have been shown to be of diagnostic and prognostic importance in oncology while offering novel therapeutic approaches. In more recent years, the role of epigenetics in oropharyngeal cancer has gained appreciation with new opportunities for translational research. The purpose of this review is to provide a general overview of epigenetic mechanisms in oropharyngeal squamous cell carcinoma (OPSCC) and knowledge of how this could be applied to novel treatment strategies.

## Main text

### HPV, epigenetics and oropharyngeal cancer

The incidence of HNSCCs has been gradually decreasing since the 1980s in association with the declining use of tobacco products. However, the incidence of OPSCC been steadily increasing over the past decade due to the rise in oncogenic HPV infections [13–16]. Early studies suggested HPV-positive tumors accounted for approximately 40–60% of OPSCCs [15, 17]. These rates have risen to greater than 80% in some regions [14, 18–24].

HPV is a nonenveloped, double-stranded DNA virus that is approximately 8000 base pairs (bp_ [25]. Over 150 serotypes of HPV have been associated with human epidermal and mucosal epithelium. HPV is classified as a sexually transmitted virus, with the majority of infections transmitted human-to-human via genital-to-genital or oral-to genital contact [26–28]. Viruses are categorized as either “low-risk” or “high-risk” with regard to their oncogenic potential. Low-risk types such as HPV 6 and 11 often manifest in the form of epithelial warts or oral papillomas [29]. Approximately 13 HPV serotypes are included in the high-risk category. Serotypes 16, 18, 31, and 33 have causal links with HPV-associated oropharyngeal carcinogenesis; however, HPV-16 has shown a much higher association with OPSCC (>90%) relative to other serotypes [13, 30–36]. Their viral genome consists of eight proteins (E1, E2, E4, E5, E6, E7, L1, L2) involved in viral replication, maintenance, and capsid structure [37, 38]. Viral proteins E6 and E7 have been shown to play an important role in carcinogenesis.

Upon infection of the host cell, HPV replicates its genome as extrachromosomal elements within the nucleus and integrates into the host genome. In cancer cells, chromosomal integration results in an increased expression and stabilization of viral oncoproteins E6 and E7 [39]. HPV integration sites are broadly distributed throughout the human genome, with various serotypes focusing on specific regions. HPV-16 favors integration at chromosomes 1, 2, 3, 5, 8, and 9 [40]. An interesting discovery by Akagi et al. has shown an association between the number of HPV integrants and their effects on neighboring gene expression. They found a higher number of integrants resulted in the direct disruption of neighboring genes via alterations in genomic structures [41]. This area of research is still in its infancy and further investigation into HPV integration-associated mutagenesis is required.

HPV-positive OPSCCs have distinct host gene expression profiles relative to HPV-negative OPSCCs [42–47]. These gene expression differences are thought to involve mechanisms of cancer tumorigenesis, proliferation, invasion, and metastasis. These differences are further reflected by distinct clinical presentations and responses to treatment modalities [13, 14, 17, 21, 48–57]. HPV-positivity is most often determined clinically by p16 overexpression, as an acceptable surrogate marker of this disease. The impact of p16 for diagnostics, prognostics and treatment stratification of OPSCC has highlighted the clinical utility of biomarkers for this disease [57–61]. In other cancers, novel epigenetic biomarkers have shown an increase in popularity for their potential specificity and biomarker-directed therapy [62].

DNA methylation, histone modifications and miRNA modifiers have all been shown to be important biomarkers and their role in OPSCC will be discussed in this review (Table 1). Specific DNA methylation patterns are showing increased promise as biomarkers, with some investigators some claiming superiority to other markers, as they hold higher levels of stability and can be amplified in a cost-effective manner [63, 64] Histone modifications may also have potential utilization as prognostic markers. For example, the methyltransferase enhancer of zeste homolog 2 (EZH2) and its substrate (H3K27 methylation) is overexpressed in numerous cancer types and is frequently indicative of a poor prognosis [7, 58, 65, 66]. However, histone modifications may be limited as biomarkers in isolation without knowing the extent of gene activity changes are associated with [7]. Despite being the most recently discovered of the epigenetic modifiers, miRs have shown some of the greatest potential as prognostic markers. MiRs have been shown to play central roles in tumorigenesis, invasion, metastasis, and responses to therapy [7, 67].

**Table 1:** Epigenetic regulators specific to OPSCC

Name	Description	Role in OPSCC	Reference
Histone Modifying Proteins			
EZH2	PRC2 protein	Hypermethylation of H3K27me3	[90]
BMI1	PRC1 protein	Stabilization of H3K27me3	[90]
DNA Methylation			
DNMT1	DNA methyltrasferase	Overexpression	[145]
DNMT3A	DNA methyltrasferase	Overexpression, *de novo* methylation	[62, 145, 146]
ncRNAs^a^			
miR-21	microRNA	Overexpression	[145]
miR-205	microRNA	Overexpression	[145]
miR-181	microRNA	Overexpression	[146, 147]
miR-17–92 cluster	microRNA	Overexpression	[62, 148]
miR-106b–25 cluster	microRNA	Overexpression	[58, 149]
miR-106–363 cluster	microRNA	Overexpression	[62]
Let-7d	microRNA	Downregulation	[66]

^a^ *Compared to normal tissues*, *only miRs frequently associated with cancer diagnosis*; *EZH2* Enhancer of zeste 2 polycomb repressive complex 2 subunit, *DNMT* DNA methyltransferase, *BMI1* B-cell–specific Moloney murine leukemia virus integration site 1

### DNA methylation in oropharyngeal cancer

Alterations in DNA methylation occur via three mechanisms; hypomethylation, hypermethylation, and loss of imprinting [68, 69] Hypomethylation of gene promoter regions can result in the activation of various proto-oncogenes and chromatin restructuring [70]. DNA hypermethylation tends to be site-specific, targeting promoter CpG islands catalyzed by a set of enzymes known as DNA methyltransferases (DNMTs). There are three primary DNMTs; DNMT1, responsible for the maintenance of the standard epigenome, DNMT3a and DNMT3b, responsible for *de novo* methylation patterns [71–73]. DNA hypermethylation in cancers often results in the silencing of various genes, frequently tumor suppressors involved in cell cycle control, DNA repair mechanisms, and apoptosis [1, 74–76].

The most well documented epigenetic event occurs directly at the level of DNA, with 5’ methylation of CpG residues, primarily at gene promoter regions. In OPSCC, distinct host methylation profiles can be seen in HPV-positive cancers when compared to HPV-negative cancers [47, 77]. Nearly three times as much differentiation in methylation profiles can be seen between HPV-positive and HPV-negative disease when compared to adjacent somatic cells [78]. HPV-positive cancers have been found to have higher levels of methylation in specific regions of the genome (promoters, genic, and LINE-1). HPV-negative cancers show a much higher degree of genome-wide hypomethylation. It has been suggested that-negative cancers are far less genomically stable relative to their HPV-positive counterparts [47, 72, 79]. Genomic instability in turn leads to widespread deregulation of cellular processes characteristic of aggressive tumors.

**Fig. 1:**
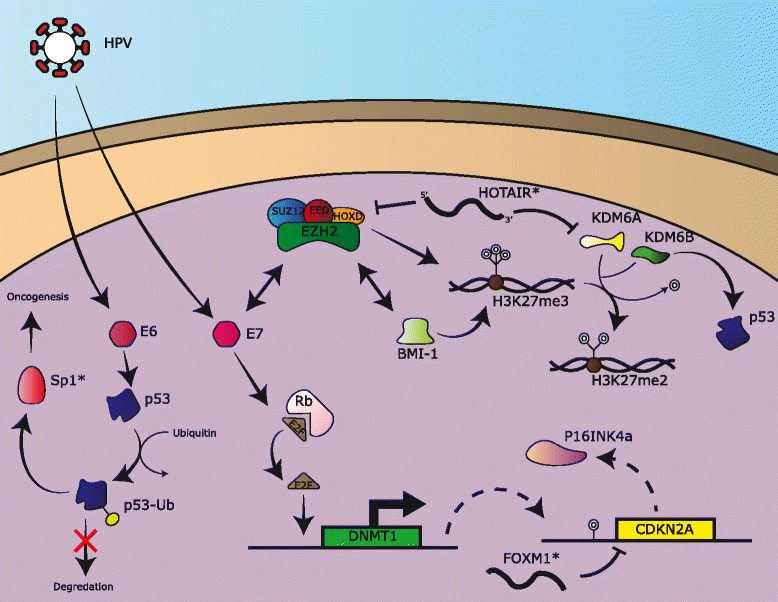
Summary of epigenetic pathways involved in oropharygeal squamous cell carcinoma. Oncogenic human papillomavirus integrated into the human genome, resulting in the expression of HPV-associated proteins E6 and E7. This results in alterations of p53, Rb and Polycomb Repressive Complex (shown here including EZH2, SUZ12, EED and HOXD) related pathways with downstream epigenetic deregulation in OPSCC. Overexpression of P16INK4a occurs as a result of loss of Rb and is used as clinical surrogate marker for HPV-positive OPSCC. *FOXM1 and HOTAIR are presumed to have a role in OPSCC based on studies in OCSCC

DNMT dysregulation is one potential mechanism for altered DNA methylation in OPSCCs. HPV-positive HNSCCs have shown increased expression in DNMT1 and DNMT3a, a pattern also seen in cervical cancers, suggesting a common mechanism of carcinogenesis by HPV [47, 80, 81]. This process is known to occur through HPV viral oncoproteins E6 and E7 (Fig. 1). HPV viral oncoprotein E6 causes the inhibition of the p53 tumor suppressor protein [48, 72, 82, 83]. As suggested by Anayannis et al. [82], this inhibition allows transcription factor Sp1 to be overexpressed to promote oncogenesis. The more notable interaction is seen by HPV E7 as it has been shown to directly interact with the tumor suppressor pRb, allowing the release of E2F (Fig. 1) from its protein complex to promote the transcription of DNMT1 [84]. E7 has also been shown to directly interact with DNMT1 in vitro, however, its implication requires further investigation [85].

Low expression of *CDKN2A* is seen in HPV-negative cancers, while high expression is found in HPV-associated disease [86]. Schlecht et al. has identified four *CDKN2A* loci downstream of the p16INK4A and p14ARF transcription start sites that are frequently hypermethylated in HPV-positive OPSCC, suggesting a potential mechanism for p16 overexpression in HPV-positive OPSCC [81]. This study also identified multiple Sp1 binding sites within the *CDKN2A* locus, further supporting the pRb/E2F pathways role in carcinogenesis (Fig. 1).

### Histone post-translational modifications in oropharyngeal cancer

The structure of chromatin is dynamic and involves numerous pathways that regulate cell metabolism. The most basic unit of chromatin is the nucleosome, a 165 bp strand of DNA wrapped in a left-handed supercoil around an octamer core of histones approximately 1.7 turns. The octamer core consists of two of each four globular proteins; H2A, H2B, H3, and H4, with a singular histone (H1) providing linkage alongside DNA [72, 87]. At the amino-terminal ends of the histone proteins, various post-translational modifications can be applied. Through the modification of histone structures, gene expression is regulated via the allowance or blockage of access to various target genes to transcriptional machinery. These include acetylation, phosphorylation, methylation, ubiquitination, sumoylation, and ADP-ribosylation. These modifications, while all able to remodel chromatin structure, frequently show aberrations in acetylation and methylation profiles in human cancers [88–91]. The primary modulators of histone methylation and acetylation processes include histone methyltransferases (HMTs), histone demethylases (HDMs), histone acetyltransferases (HATs), and histone deacetylases (HDACs) [11, 12].

There are two known types of HATs; A-type within the nucleus, involved in the catalysis of transcription-related acetylation, and B-type within the cytosol, associated with newly generated histones. HATs facilitate the opening of chromatin for recruitment of transcriptional machinery by transferring acetyl groups from acetyl-CoA to specific lysine residues [92, 93]. Their overexpression has been associated with various cancers by aberrantly driving gene expression [94, 95]. Conversely, the overexpression of HDACs promotes deacetylation, resulting in abnormal gene silencing in the cancer epigenome [95–97].

Histone methylation works in a comparable process, where methylation, or multiple methylations, of lysine and arginine residues will result in structural rearrangements of chromatin [91, 92]. Like histone acetylation, aberrant expression of histone HMTs (enzymes that add methylation) and HDMs (enzymes that remove methylation) have been associated with carcinogenesis of various cancers [98–104]. Tumor cells frequently show altered methylation profiles on histone H3 at specific lysine sites including K4, K9, K27, K36, K79 and on histone H4 K20 [104–110]. Adding further complexity to this process, mono-, di- or trimethylation can occur on any given histone methylation site.

A recurring histone modifier in cancer literature is the methyltransferase EZH2, a catalytic subunit within the polycomb repressive protein complex 2 (PRC2) [101]. EZH2 catalyzes the trimethylation of lysine 27 on histone 3 (H3K27me3) and appears to have a regulatory role in cell proliferation and cell-cycle progression. Various cancers have displayed overexpression of EZH2 and it has been associated as a marker for malignancy potential and poor clinical prognosis, including HPV-positive OPSCC [111, 112]. HPV status and EZH2 overexpression are closely related, as *EZH2* is a downstream target of E7 in vitro via the release of E2F from pocket proteins (Fig. 1) [113]. As proposed by Holland et. al., p53 suppression via E6 may also provide a mechanism for EZH2 overexpression [113]. As expected, HPV-positive (positive for p16INK4A) OPSCCs have genome-wide elevations of H3K27me3 [103]. An additional method of carcinogenesis by EZH2 overexpression may be through DNMT3A, as EZH2 has been shown to recruit DNMT3A; however, the *de novo* functionality of DNMT3A is not directly activated by this process [111].

B-cell–specific Moloney murine leukemia virus integration site 1 (BMI1) is a central component of the polycomb repressive complex 1 (PRC1). Overexpression has also been associated in carcinogenesis, functioning by stabilizing H3K27me3 and preventing transcriptional initiation [112, 114, 115]. Huber et al. have shown that BMI1 expression plays a potential role as a prognostic biomarker of OPSSC. Its aberrant expression in conjunction with p16 silencing is negatively correlated with recurrence-free survival in OPSCC [112].

### Small and non-coding RNAs in oropharyngeal cancer

ncRNAs have been implicated in carcinogenesis and malignancy progression, with one of the first examples shown in chronic lymphocytic leukemia [116]. ncRNA are categorized based on their size. Nucleic acids less than 200 bp are known as small ncRNAs and greater than 200 bp are known as long non-coding RNAs (lncRNAs) [117]. Included within the small ncRNAs are the small interfering RNAs (siRNAs), micro RNAs (miRs), and PIWI-interacting RNAs (piRNAs) [118]. The majority of cancer research focuses on miRs as they have been shown to promote carcinogenesis through multiple pathways, including the direct interaction with mRNA, either through mRNA translation inhibition or mRNA degradation [67, 119]. Epigenetic silencing of specific miRs may have a causal link in carcinogenesis as they have shown to act as tumor suppressors. lncRNAs have no formal categorization. Most are organized based on the transcripts function; chromatin remodelling and transcription factor modulation. The majority of cancer literature focusing on the former, such as the well *Xist* transcript [6, 120].

Our current knowledge of the ncRNAs role in carcinogenesis is relatively limited, largely due to the novelty of the molecules discovery. The direct implication ncRNAs in OPSCC further confirm this, as known ncRNAs involved are limited to a few products. For the purposes of this section, the field of study will be expanded slightly to include ncRNAs implicated in other head and neck cancers, in addition to those found in OPSCC.

HPV status in tumors has shown distinct epigenetic profiles and clinical relevance. These distinctions is well outlined in the review by Lajer et al., who compared epigenetic profiles of HPV-positive cervical and head and neck cancers. They found a significant overlap in various miR clusters [121]. Sethi et al. outlined a comprehensive list of multiple miRs with aberrant expression patterns in head and neck cancers in addition to those mentioned by Lajer and colleagues [122]. This suggests distinct miR expressions are associated with HPV-associated cancers. This concept is further enforced by the direct interaction of some miRs, such as miR-15 and miR-16, with viral E6 and E7 [123].

One miR not acknowledged in literature, but which provides great interest, is miR-101. miR-101’s aberrant expression, namely its downregulation, has been involved in multiple cancers and has shown to mediate the overexpression of EZH2 [124–126]. The restoration of miR-101 via DNMT3A inhibition has also been shown to suppress lung tumorigenesis [126]. As both DNMT3A and EZH2 overexpression occurs in HPV-positive OPSCC, it may serve an important role in carcinogenesis [82].

Of the ncRNAs present within head and neck cancer literature, lncRNAs mirror the scarcity of miR counterparts. However, one lncRNA in particular, HOTAIR, has shown great promise as a potential biomarker. HOTAIR is a non-coding RNA transcript of 2.2 kb transcribed from the HOXC locus to transcriptionally silence HOXD [117, 127]. Interactions of HOTAIR have shown the 5’domain to bind to the PRC2 complex described previously as well the 3'domain binding the histone demethylase KDM1A (Fig. 1). These interactions potentially show methods of carcinogenesis, as its overexpression has been demonstrated in multiple cancer types including esophageal, nasopharyngeal, breast, pancreatic, and colorectal cancers [127–129] Overexpression of HOTAIR has been associated with an overall poor clinical prognosis, demonstrating increased lymph node metastasis and resistance to apoptosis. HOTAIR’s direct linkage to OPSCC requires further study. Other lncRNAs of interest are FTH1P3, PDIA3F and GTF2IRD2P1, as they have been associated with the progression and metastasis of oral SCC via the targeting of multiple tumor regulator genes [130].

### Epigenetic chemotherapeutics

Aberrant events within the epigenome are suggested to occur more readily than structural gene modification through mutation. Given the reversible nature and specificity of epigenetic modifications, they have become an attractive target for cancer prevention and therapeutic intervention [2, 5, 73, 131]. Epigenetic chemotherapeutics are classified into two primary classes; histone deacetylase (HDAC) inhibitors and DNMT inhibitors. These classes are likely to expand as our knowledge of epigenetics advances and further chemotherapeutics are developed and tested. There are currently five USFDA-approved epigenetic chemotherapeutics on the market. Two are DNMT inhibitors; 5-acactidine (Vidaza) and 5-aza-2’-deoxycitidine (Decitabine). Three are HDAC inhibitors; suberoylanilide hydroxamic acid (Vorinostat), F-228 (Romidepsin), and LAQ-824 (Farydak). Current epigenetic chemotherapeutics in clinical trials or approved by the USFDA are summarized in Table 2.

**Table 2:** Potential epigenetic chemotherapies for oropharyngeal carcinoma

Chemotherapeutic Agent	Status	Reference
DNMT inhibitors		
Arsenic trioxide	Clinical Trials	[112]
5- azacytidine (Vidaza, Celgene)	USFDA Approved	[47]
5-aza-2′-deoxycitidine (Decitabine, Dacogen, SuperGen)	USFDA Approved	[47]
MG98	Clinical trials	[122]
HDAC inhibitors		
LAQ-824/LBH 589 (Farydak, panobinostat)	USFDA Approved	[122]
PXD-101(Belinostat)	Clinical trials	[122]
Valproic acid (Mg valproate)	Clinical trials	[122]
Suberoylanilide hydroxamic acid (vorinostat, SAHA)	USFDA Approved	[122]
FK-228 (romidepsin)	USFDA Approved	[121]
Phenylbutyrate	Clinical trials	[122]
MS-275 (entinostat)	Clinical trials	[150, 151]
CI-994	Clinical trials	[90, 120]
MGCD-0103 (Mocetinostat)	Clinical trials	[145]
JNJ-26481585 (Quisinostat)	Clinical trials	[146, 147]
HMT inhibitors		
EPZ-6438 (E7438, Epizyme)	Clinical trials	[62, 148]
3-Deazaneplanocin (DZNep)	Clinical trials	[58]
EPZ-5676	Clinical trials	[62]
EPZ-5687	Preclinical	[58]
GSK-343	Preclinical	[58]

*DZNep*, 3-deazaneplanocin A; *USFDA* United States Food and Drug Association

Both DMNT inhibitors, Vidaza and Decitabine, are the only epidrugs that have been approved for the treatment of patients with acute myeloid leukemia (AML) and myelodyplastic syndrome (MDS) [132]. Vidaza and Decitabine are nucleoside analogs of cytosine modified in position five of their pyrimidine ring [133]. Upon exposure, Vizdaza is incorporated into RNA and Decitabine is incorporated into DNA where they disrupt interactions between DNMTs and DNA. During this process, a covalent bond is formed with DNMT triggering a DNA damage signal and targeting the DNMT for degradation. When utilized in clinical practice, their applicability encountered major limitations. These are characterized by poor bioavailability, poor activity with solid tumors, severe toxic effects, instability in physiological media, and gross non-specific changes to epigenome to both normal and cancer cells. Fortunately, several new specific inhibitors are under development. Of these are [133] MG98, small molecule RG108, nucleoside analog Zebularine, and arsenic trioxide. These inhibitors have shown increased specificity, chemical stability, increased bioavailability, and lower cytotoxic effects [132, 133].

HDAC inhibitors are regularly divided into four different groups based on their chemical structure. These are hydroximates, cyclic peptides, aliphatic acids, and benzamides. Within the hydroximate class are two USFDA approved agents, Vorinostat and the newly approved Farydak, as well as JNJ-26481585 (Quisinostat) currently in clinical trials [134–136]. Aliphatic acids contain three agents currently in clinical trials; valproic acid, phenylbutyrate, and Belinostat. Cyclic peptides contain the USFDA approved Romidepsin and benzamides contain three agents in the clinical trial stages; MS-275 (entinostat), MGCD-0103 (Mocetinostat), CI-994 [131, 135]. The mechanisms of HDAC inhibitors are not fully understood, but are thought to alter gene expression via regulation at both epigenetic and post-translational modification levels [137, 138]. Evidence also suggests HDAC inhibition may alter tumor progression by inhibiting tumor angiogenesis [138]. HDAC inhibitors are well tolerated relative to other epigenetic chemotherapeutics. However, these drugs still display poor activity against solid tumors when utilized on their own. Suggested application is specific timing in conjunction with current chemotherapeutics [139, 140] 4. Another novel histone modifier inhibitor in the clinical trial stage is EPZ-6438 (Epizyme), an inhibitor of histone methyltransferase DOT1L [141–143]. While still requiring further study for conclusive data, initial studies suggest its efficacy and tolerance.

The use of miR’s as potential targets for chemotherapeutics is still in its infancy. Multiple studies have shown the significant effects of upregulation and downregulation of specific miRs on cancer. Of note is miR-21 s direct role in tumorigenesis following upregulation and reduced tumor survival and progression following its downregulation [144].

## Conclusions

As with other cancers, epigenetics has a fundamental role in the pathophysiology of OPSCC. HPV positive and negative OPSCCs have distinct epigenetic profiles, consistent with their pathological and clinical differences. An understanding of epigenetics in OPSCC provides opportunities for the discovery and application of novel biomarkers and treatments.

## Abbreviations

BMI-1: B-cell–specific Moloney murine leukemia virus integration site 1
Bp: Base pair
DNA: Deoxyribonucleic acid
DNMT: DNA methyltransferase
DZNep: 3-deazanoplanocin
EZH2: Enhancer of zeste homolog 2
HAT: Histone acetyltransferase
HDAC: Histone deacetylase
HDM: Histone demethylase
HMT: Histone methyltransferase
HPV: Human papillomavirus
lncRNA: Long non-coding RNA
miRNA: micro RNA
ncRNA: Non-coding RNA
piRNA: PIWI-interacting RNA
PRC: Polycomb repressive complex
RNA: Ribonucleic acid
siRNA: Small interfering RNA
USFDA: United States Food and Drug Association
